# Detailed balance for diffusion in a potential with trapping and forward–backward symmetry of trapping time distributions

**DOI:** 10.1063/1.5142566

**Published:** 2020-06-08

**Authors:** Alexander M. Berezhkovskii, Dmitrii E. Makarov

**Affiliations:** 1 Mathematical and Statistical Computing Laboratory, Office of Intramural Research, Center for Information Technology, National Institutes of Health, Bethesda, Maryland 20892, USA; 2 Department of Chemistry, University of Texas at Austin, Austin, Texas 78712, USA; 3 Oden Institute for Computational Engineering and Sciences, University of Texas at Austin, Austin, Texas 78712, USA

## Abstract

For particles diffusing in a potential, detailed balance guarantees the absence of net fluxes at equilibrium. Here, we show that the conventional detailed balance condition is a special case of a more general relation that works when the diffusion occurs in the presence of a distributed sink that eventually traps the particle. We use this relation to study the lifetime distribution of particles that start and are trapped at specified initial and final points. It turns out that when the sink strength at the initial point is nonzero, the initial and final points are interchangeable, i.e., the distribution is independent of which of the two points is initial and which is final. In other words, this conditional trapping time distribution possesses forward–backward symmetry.

Consider a particle diffusing along a one-dimensional coordinate *x* in a constraining potential *U*(*x*), ${\left.U\left(x\right)\right|}_{\left|x\right|\to \infty }\to \infty$. Its propagator (Green’s function), denoted by *G*(*x*, *t*|*x*_0_), is the probability density of finding the particle at point *x* at time *t*, conditional on that the particle started from *x* = *x*_0_ at *t* = 0. The propagator satisfies the Smoluchowski equation,

$$\frac{\partial G}{\partial t}=D\frac{\partial }{\partial x}\left[e^{-\beta U\left(x\right)}\frac{\partial }{\partial x}\left(e^{\beta U\left(x\right)}G\right)\right],$$ (1)

subject to the initial condition *G*(*x*, 0|*x*_0_) = *δ*(*x* − *x*_0_). In Eq. (1), *D* is the particle diffusivity, and *β* = 1/(*k*_*B*_*T*), where *k*_*B*_ and *T* are the Boltzmann constant and absolute temperature. This propagator describes the relaxation of the initial *δ*-distribution to the equilibrium one,

$$\underset{t\to \infty }{\text{lim}}G\left(x,t|x_{0}\right)=p_{eq}\left(x\right)=e^{-\beta U\left(x\right)}/\int_{-\infty }^{\infty }e^{-\beta U\left(x\right)}dx.$$ (2)

Once *G*(*x*, *t*|*x*_0_) is known, the time evolution of any initial distribution *p*(*x*_0_, 0) can be determined by computing the convolution of the two functions.

The propagator satisfies the detailed balance condition,

$$G\left(x,t|x_{0}\right)e^{-\beta U\left(x_{0}\right)}=G\left(x_{0},t|x\right)e^{-\beta U\left(x\right)},\;t\geq 0,$$ (3)

which is a relationship between the propagators *G*(*x*, *t*|*x*_0_) and *G*(*x*_0_, *t*|*x*) that guarantees that there are no net fluxes between arbitrary chosen points *x* and *x*_0_ at equilibrium. Ultimately, detailed balance is a consequence of time reversibility of diffusion trajectories contributing to the propagator.^1–4^

When particle diffusion occurs in the presence of an arbitrary sink *γ*(*x*), *γ*(*x*) ≥ 0, Eq. (1) takes the form:

$$\frac{\partial G}{\partial t}=D\frac{\partial }{\partial x}\left[e^{-\beta U\left(x\right)}\frac{\partial }{\partial x}\left(e^{\beta U\left(x\right)}G\right)\right]-\gamma \left(x\right)G.$$ (4)

Here, the propagator vanishes as *t* → ∞, since the particle is trapped by the sink,

$$\underset{t\to \infty }{\text{lim}}G\left(x,t|x_{0}\right)=0.$$ (5)

Although there is no equilibrium in this case, the propagator nevertheless satisfies the detailed balance condition [Eq. (3)]. This is one of the main results of this work.

We use this result to show that the trapping time distributions possess forward–backward symmetry. To explain what this symmetry is, consider an ensemble of particles starting from *x* = *x*_*i*_, where the sink strength is not equal to zero, *γ*(*x*_*i*_) > 0. Some of the particles are trapped by the sink *γ*(*x*) at a point *x* = *x*_*f*_. We use these ensemble members to find the distribution of their lifetime, denoted by $p_{x_{i}\to x_{f}}\left(t\right)$. We can repeat this procedure for an ensemble of particles starting from *x* = *x*_*f*_ and find the lifetime distribution of the ensemble members trapped at the point *x* = *x*_*i*_, denoted by $p_{x_{f}\to x_{i}}\left(t\right)$. It turns out that these trapping time distributions are identical,

$$p_{x_{i}\to x_{f}}\left(t\right)=p_{x_{f}\to x_{i}}\left(t\right),$$ (6)

and hence possess forward–backward symmetry. This is another main result of this work. To avoid confusion, it is worth noting that the trapping time distributions differ from the conditional first passage time distributions discussed, e.g., in Ref. 5.

The equality in Eq. (6) is applicable for transitions between points, in which *γ*(*x*) > 0. As an example, consider channel-facilitated transport of diffusing particles between two compartments separated by a membrane. To a good approximation, particle dynamics in the channel can be described as one-dimensional diffusion along the channel axis in a potential of mean force. Particle escape from the channel is modeled by imposing radiation boundary conditions at the channel ends.^6,7^ A particle entering the channel either returns to the same compartment from which it entered or passes through the channel and escapes on the opposite side of the membrane. Formally, one can replace the radiation boundaries at the channel ends by reflecting ones and place localized (*δ*-function) sinks near them with the sink strengths equal to the trapping rates entering the radiation boundary conditions. The particle is trapped by the sink located at the channel entrance in the first of the two above-mentioned scenarios and by the sink located at the channel exit in the second scenario. For particles passing through the channel, it has been shown that the distribution of their residence time in the channel is independent of the passage direction^8–10^ and hence satisfies Eq. (6).

The passage time distribution remains finite even when the channel ends are perfectly absorbing boundaries and the passage probabilities vanish. In this limiting case, they describe distributions of the direct-transit time also referred to as the transition path time.^11^ This time, which is the duration of a trajectory fragment during which the particle diffuses from its starting point to the end point without touching the starting point, has attracted a flurry of recent theoretical^12,13^ and experimental^14,15^ efforts. It is known that the direct-transit time distributions are also independent of the passage direction.^11,16–19^ Thus, the identity of the trapping time distributions [Eq. (6)] is a generalization of known results and contains these results as special cases. The identities of the forward–backward transit time distributions discussed in Refs. 8–11 and 16–19 were proved assuming that the particle can be trapped only at the interval end points. Here, we show that this assumption can be relaxed, and the identities remain unchanged even if the particle can be trapped within the interval. As examples, we mention transport of molecular motors along microtubules and protein search for the target site on DNA,^20–22^ where the detachment from the microtubule or a DNA may be modeled by introducing a sink.

Our interest in the trapping time distributions and related problems was stimulated by recent single-molecule and single-channel experiments, in which such distributions can potentially be measured with high accuracy,^14,15,23–26^ thereby providing new insights into the mechanisms of channel transport, biomolecular folding and binding, and other biophysical phenomena.

We now prove that the propagator *G*(*x*, *t*|*x*_0_) of a particle diffusing in the presence of an arbitrary sink satisfies the detailed balance condition [Eq. (3)]. To this end, we write this propagator as

$$G\left(x,t|x_{0}\right)=e^{-\beta \left[U\left(x\right)-U\left(x_{0}\right)\right]/2}g\left(x,t|x_{0}\right).$$ (7)

Substituting this into Eq. (4), one can check that *g*(*x*, *t*|*x*_0_) satisfies a Schrödinger-like equation,

$$\begin{array}{l} \frac{\partial g}{\partial t}=Ĥg=D\frac{\partial^{2}g}{\partial x^{2}}-\left\{D\left[{\left(\frac{1}{2}\beta \frac{dU\left(x\right)}{dx}\right)}^{2}-\frac{1}{2}\beta \frac{d^{2}U\left(x\right)}{dx^{2}}\right]+\gamma \left(x\right)\right\}g, \end{array}$$ (8)

subject to the initial condition *g*(*x*, 0|*x*_0_) = *δ*(*x* − *x*_0_). Thus, *g*(*x*, *t*|*x*_0_) is Green’s function for Eq. (8), in which the Hamiltonian Ĥ is a Hermitian operator. The eigenfunction expansion (spectral representation) of *g*(*x*, *t*|*x*_0_) is given by

$$g\left(x,t|x_{0}\right)=\overset{\infty }{\underset{n=1}{\sum }}\varphi_{n}\left(x\right)\varphi_{n}\left(x_{0}\right)e^{\varepsilon_{n}t},$$ (9)

where *ε*_*n*_ (*ε*_*n*_ < 0) and *φ*_*n*_(*x*) are the eigenvalues and eigenfunctions of Ĥ,

$$Ĥ\varphi_{n}\left(x\right)=\varepsilon_{n}\varphi_{n}\left(x\right),\;n=1,2,\ldots .$$ (10)

Substituting *g*(*x*, *t*|*x*_0_) in Eq. (9) into Eq. (7), we obtain

$$G\left(x,t|x_{0}\right)=e^{-\beta \left[U\left(x\right)-U\left(x_{0}\right)\right]/2}\overset{\infty }{\underset{n=1}{\sum }}\varphi_{n}\left(x\right)\varphi_{n}\left(x_{0}\right)e^{\varepsilon_{n}t}.$$ (11)

Multiplying this by $e^{-\beta U\left(x_{0}\right)}$, we arrive at

$$\begin{array}{ll} G\left(x,t|x_{0}\right)e^{-\beta U\left(x_{0}\right)} & =e^{-\beta \left[U\left(x\right)+U\left(x_{0}\right)\right]/2}\overset{\infty }{\underset{n=1}{\sum }}\varphi_{n}\left(x\right)\varphi_{n}\left(x_{0}\right)e^{\varepsilon_{n}t}\\ & =G\left(x_{0},t|x\right)e^{-\beta U\left(x\right)}, \end{array}$$ (12)

which is the detailed balance condition for nonequilibrium systems [Eq. (3)].

Note that, although we focus on the case of a constraining potential (${\left.U\left(x\right)\right|}_{\left|x\right|\to \infty }\to \infty$), the results in Eqs. (3) and (6) remain unchanged if we relax this assumption and allow the particle to escape to infinity. In this case, the eigenvalue spectrum of the Hamiltonian Ĥ defined in Eq. (8) is continuous, and the summation in Eqs. (9), (11), and (12) should be replaced by integration. The rest of the above reasoning remains unchanged.

We illustrate our result with two examples. In the first, the sink strength is distributed uniformly along *x*, *γ*(*x*) = *γ*. Such a model may, for example, be used to describe the detachment of a molecular motor moving along its track, if it occurs with the same probability regardless of the motor position. Let *G*_0_(*x*, *t*|*x*_0_) be Green’s function in the absence of trapping, which satisfies Eq. (1). It is easy to verify that the solution of Eq. (4) in the case where the trapping rate is coordinate-independent is simply given by *G*(*x*, *t*|*x*_0_) = *e*^−*γt*^*G*_0_(*x*, *t*|*x*_0_), and since *G*_0_(*x*, *t*|*x*_0_) satisfies detailed balance [Eq. (3)] so does Green’s function *G*(*x*, *t*|*x*_0_).

The second example represents the opposite extreme, with spatially localized trapping. Specifically, we consider the following coordinate dependence of the sink strength, *γ*(*x*) = *γ*_*a*_*δ*(*x* − *a*) + *γ*_*b*_*δ*(*x* − *b*), where *δ*(*x*) is Dirac’s delta-function. Such a scenario arises, for example, when the dynamics of a particle inside a membrane channel (*a* < *x* < *b*) is considered, and sinks are introduced to describe the escape from the channel to the bulk on its either side.^6,7^ Green’s function *G*(*x*, *t*|*x*_0_) of such a system can be related to Green’s function *G*_0_(*x*, *t*|*x*_0_) in the absence of trapping using the Dyson equation—see, e.g., Ref. 27,

$$G\left(x,t|x_{0}\right)=G_{0}\left(x,t|x_{0}\right)-\overset{t}{\underset{0}{\int }}dt^{\prime }\overset{\infty }{\underset{-\infty }{\int }}dx^{\prime }G_{0}\left(x,t-t^{\prime }|x^{\prime }\right)\gamma \left(x^{\prime }\right)G\left(x^{\prime },t^{\prime }|x_{0}\right).$$ (13)

We note that the Dyson equation is more familiar in the context of quantum mechanics, where it can be used to describe the propagator of a time-dependent Schrödinger equation in the presence of a perturbation, but it can be analogously derived for the propagator (i.e., Green’s function) of Eq. (4), where the sink term plays the role of a perturbation. Introducing a Laplace-transformed Green’s function, $Ĝ\left(x,s|x_{0}\right)=\overset{\infty }{\underset{0}{\int }}dte^{-st}G\left(x,t|x_{0}\right)$, the Dyson equation can be rewritten, for the two-delta-function-sink model, as

$$Ĝ\left(x,s|x_{0}\right)={Ĝ}_{0}\left(x,s|x_{0}\right)-\gamma_{a}Ĝ\left(x,s|a\right){Ĝ}_{0}\left(a,s|x_{0}\right)-\gamma_{b}Ĝ\left(x,s|b\right){Ĝ}_{0}\left(b,s|x_{0}\right).$$ (14)

Applying this result to *x* = *a*,*b* and *x*_0_ = *a*,*b*, it can be shown that

$$Ĝ\left(b,s|a\right)=\frac{{Ĝ}_{0}\left(b,s|a\right)}{\left[1+\gamma_{a}{Ĝ}_{0}\left(a,s|a\right)\right]\left[1+\gamma_{b}{Ĝ}_{0}\left(b,s|b\right)\right]-\gamma_{a}\gamma_{b}{Ĝ}_{0}\left(a,s|b\right){Ĝ}_{0}\left(b,s|a\right)},$$ (15)

and a similar expression is obtained for Ĝ(a,s|b) by simply exchanging *a* and *b*. Unlike the case of the coordinate-independent sink strength, the temporal behavior of Green’s functions in the case of two localized sinks is nontrivial and depends on both *γ*_*a*_ and *γ*_*b*_, as well as on the sink locations. Yet when taking a ratio Ĝ(a,s|b)/Ĝ(b,s|a), we observe that it is identical to the ratio ${Ĝ}_{0}\left(a,s|b\right)/{Ĝ}_{0}\left(b,s|a\right)=\text{exp}\left\{-\beta \left[U\left(a\right)-U\left(b\right)\right]\right\}$, which proves the detailed balance condition in the presence of trapping for this model.

To prove the forward–backward symmetry of the trapping time distributions, consider a particle starting from *x* = *x*_*i*_, where *γ*(*x*_*i*_) ≠ 0, at time *t* = 0. Let $q_{x_{i}\to x_{f}}\left(t\right)$ be the flux trapped by the sink *γ*(*x*) at point *x* = *x*_*f*_ at time *t*,

$$q_{x_{i}\to x_{f}}\left(t\right)=\gamma \left(x_{f}\right)G\left(x_{f},t|x_{i}\right).$$ (16)

The integral of this flux over time from zero to infinity, denoted by $\Phi_{x_{i}\to x_{f}}$,

$$\Phi_{x_{i}\to x_{f}}=\int_{0}^{\infty }q_{x_{i}\to x_{f}}\left(t\right)dt=\gamma \left(x_{f}\right)\int_{0}^{\infty }G\left(x_{f},t|x_{i}\right)dt,$$ (17)

is the spatial distribution of the location of the trapping point normalized to unity,

$$\int_{-\infty }^{\infty }\Phi_{x_{i}\to x_{f}}dx_{f}=1.$$ (18)

The time distribution for *x*_*i*_ → *x*_*f*_ trapping, denoted by $p_{x_{i}\to x_{f}}\left(t\right)$, is the flux $q_{x_{i}\to x_{f}}\left(t\right)$ normalized to its integral over time $\Phi_{x_{i}\to x_{f}}$,

$$p_{x_{i}\to x_{f}}\left(t\right)=\frac{q_{x_{i}\to x_{f}}\left(t\right)}{\Phi_{x_{i}\to x_{f}}}=\frac{G\left(x_{f},t|x_{i}\right)}{\int_{0}^{\infty }G\left(x_{f},t|x_{i}\right)dt}.$$ (19)

Correspondingly, the time distribution for *x*_*f*_ → *x*_*i*_ trapping, denoted by $p_{x_{f}\to x_{i}}\left(t\right)$, is given by

$$p_{x_{f}\to x_{i}}\left(t\right)=\frac{q_{x_{i}\to x_{f}}\left(t\right)}{\Phi_{x_{i}\to x_{f}}}=\frac{G\left(x_{i},t|x_{f}\right)}{\int_{0}^{\infty }G\left(x_{i},t|x_{f}\right)dt}.$$ (20)

Using the detailed balance condition [Eq. (3)], one can see that the trapping time distributions in Eqs. (19) and (20) are equal, as claimed in Eq. (6).

In summary, detailed balance for diffusing particles [Eq. (3)] is conventionally discussed for systems with particle conservation. Here, it is shown that Eq. (3) is true even if the particles can be trapped by a sink *γ*(*x*), and hence, the conventional detailed balance condition is a special case where *γ*(*x*) = 0. We used the detailed balance condition [Eq. (3)] in the presence of trapping to show that the trapping time distributions possess forward–backward symmetry [Eq. (6)]. Finally, we mention that the present study complements our recent work on forward–backward symmetry of transition path time distributions in nonequilibrium systems, which, in contrast to this work, is mainly focused on Markovian dynamics on networks.^28^
